# Head Injuries

**Published:** 1919-06

**Authors:** 


toms of damage to brain tissue are permanent to a certain degree. I call attention to the differentiation of merely transient symptoms from the real or permanent symptoms for the reason that I believe that if due attention is not given to same errors will be made and unnecessary and oftimes injurious operations will be undertaken.

From our observations I think it a mistake to attribute all the immediate symptoms following head injuries to destruction of brain tissue, hemorrhage or decompression due to depressed bone fragments. I am aware that many good brain surgeons advise exploratory operations in all head injuries where there is coma, shock, etc., without waiting to find out whether these symptoms will clear up within a few moments or a few hours. The result of early explanatory operations is that either too much or too little is done. Either the operation is not needed or the damage extends beyond the scope of operation. It must be remembered that a scalp wound plus a bone injury is accompanied by damage lesions which are coexistent but oftentimes independent and remote from the site of the injury. The idea of exploring every head injury is not based upon sound surgical principles and to my personal knowledge has led to many disasters. Cases without dural injury have been converted into dural cases with consequent infection. Infection is the great factor in mortality. As long as the dura is intact the intracranial structures are comparatively safe. The dura protects the brain like the skin protects the body or the peritoneum the vicera. Adhesions very quickly form the incised dura and the pia mater which are life saving.

### Operation.

The three prime objects to be obtained are:

First. The cleansing of the wound, the removal of bone fragments, foreign bodies, devitalised tissue, etc. In short the debridement of the wound.

Second. The relief of symptoms of cerebral injury.

Third. The prevention of symptoms, complications and sequellas.

The debridement is the primary object. It is perhaps the superlatively important step and must be done with exactness and expediency.

A brain debridement must be preceded by careful examination and preparation of the patient. After as careful and thorough neurological examination as well as X ray and flouroscopic examinations as it is possible to make the patient is prepared for the operation.

A preliminary hypodermic is given 1/4 gr. morphine. A very important step is the preparation of the field of operation. This should be done by either the surgeon or some one trained to handle head injuries.

The entire head must be shaven. Care must be exercised in handling head cases with brain injury especially patients with retained foreign bodies.

The field is best painted with 1/2 per cent Tr. Iodine.

Scalp injuries not involving the brain are best done under general anesthesia. If reasonably sure of surgical cleanliness the edges of the wound may be incised, by cutting perpendicularly to the skull with a very sharp knife, removing just enough of the scalp to get all the devitalized tissue, then suturing with cat gut or silk worm gut, controlling all bleeding. Infected wounds are drained, partly closed or treated open as indicated by the condition of the wound.

Injuries involving the skull or brain or both are best done under local anesthesia.

Using one-fourth to one-eighth per cent novocaine with 10 minims adrenalin to the ounce a circular block injection is made surrounding the field of approach. If we are dealing with compound comminuted fracture the work will be done through the wound. The edges of the wound are trimmed as noted above and enlarged as needed. Now comes the important part, the dealing with the bone. To properly debride a bone injury requires expertness and technical dexterity. In all penetrating wounds and depressed fractures not too very large it is far better to do block removal. This is easily done under the local anesthetic. By use of a small trephine or Hudson's cranial drill four openings are made. With the Devilbis skull cutting forceps the openings are connected up and the pieces lifted out. All small fragments and spiculae are removed. Next comes attention to the dura. If the dura has been entered, we proceed through the wound. The edges are carefully trimmed away, not destroying any more tissue than absolutely necessary. The opening may be enlarged as room is required to explore and debride the brain.

With a soft rubber catheter the wound in the brain is gently explored. If there is a F. B. its approximate position is known from the X ray findings and its general direction is indicated by the wound tract. If it is felt by the catheter, a long forcep is gently placed on it and withdrawn. The rule for removing the F. B. from the brain may be stated here. A general rule to follow is to remove the F. B. unless in doing so brain tissue will be destroyed which is not already devitalized. It is better to leave the F. B. than to destroy good brain tissue in removing it.

Macerated and soiled brain tissue is then removed, using a soft rubber catheter and a suction bulb. If all the infested or deviatalized tissue can not thus be gotten out the edges of the tissue may be trimmed with a sharp knife and the particles removed with the suction apparatus.

The wound is closed or left open and drained according to indications. In cases where the debridement has been complete and the wound is not but a few hours old many cases may be closed. If possible the dura is sutured with 00 catgut. The bone block is not replaced. The periosteum is brought over the wound in the skull if possible and the soft parts closed with silk or silk worm gut. Primary suture is a proceedure which must be carefully judged. Some careful operators report many favorable cases.

If thought best to drain, the most satisfactory method of which I have had opportunity to judge is by use of small rubber tissue drains, making a cofferdam around the edges of the incised dura. Such method favors the formation of adhesions between the dura and the arachnoid and pia mater as well as taking care of the drainage. They must be withdrawn at the end of not less than forty-eight hours. They should be replaced with iodoform or moist sterile gauze lightly packed in the wound down to the brain tissue. This drainage is gradually withdrawn as the wound heals. Silkworm gut sutures may be placed in the scalp to facilitate delayed closure, for many drainage cases may be closed at the end of five or six days.

Many methods of drainage have been advocated but we will not here enter into a discussion of their merits or defects.

### Effort to Relieve Symptoms.

These may be noted as follows:

Loss of consciousness, coma, headache, slowness of pulse, embarrassment of respiration, occular disturbances, vomiting, symptoms of hemorrhage, paralysis, convulsions, alterations of reflexes, etc. If timely and adequate operative proceedures are carried out these symptoms may be relieved except insofar as they may be due to permanent destruction of brain tissue.

Next comes the prevention of symptoms, complications and sequellas.

From this standpoint we operate to prevent:

First. Intracranial pressure. However it must be borne in mind that some cases of intracranial pressure are due to acute oedema contusions, etc., of such a nature that operation is not indicated.

Second. Progressive hemorrhage. This is prevented by thorough debridement and the control of bleeding vessels.

Third. Hernia of the brain.

Fourth. Epilepsy; a remote sequella must not be forgotten.

I briefly call attention to the value of spinal puncture both as a diagnostic aid and as a measure to relieve intracranial pressure. The spinal manometer is a very useful aid. It may be possible to establish the relative ratio of intracranial pressure to the blood pressure.

The normal spine pressure is about 5 to 7 mercurial mm. equivalent to from 60 to 100 mm. water. In patients with intracranial pressure, whatever may be the cause, the spinal pressure will give a fair index to it. The spinal pressure in increased with the increase of the blood pressure, and it must be considered in regard to its ratio to the blood pressure.

Some pretty good rules have been worked out relative to the question of the time to operate or not operate based upon a study of the spinal fluid pressure. I have not had sufficient observation or experience on that subject to venture an opinion.

I will before passing from the subject however warn of the danger of spinal puncture in cases where there is clinical evidence of great intracranial pressure. I might say rather warn against the spinal puncture and the letting out of too much fluid. I have seen death occur immediately following spinal puncture due to the jamming of the cerebrum into the foramin magnum. I have also seen patients die a few hours following spinal puncture from cerebrum involvement.

I hereby append a classification of the head injuries named in the numerical order as they occured during our service in the Evacuation Hospitals during the Argonne-Meuse offensive.

First. Fissured fractures with depression of the inner table but without grave defect of bone and without dural penetration.

Second. Simple and compound penetrating wounds.

Third. Through and through wounds.

Fourth. Wounds with retained F. B.

Fifth. Tangential wounds or fractures.

Sixth. Wounds of the sinuses.

Seventh. Compound comminuted fractures with destruction and loss of brain tissue.

Eighth. Depressed fractures with injury to the dura.

Ninth. Contrecou fractures with extra or subdural hemhorrhage.

Tenth. Intracranial hemorrhage without fracture.

Eleventh. Linear fractures extending into the foramin.

Different varieties of these fractures occurred.

Of course operative tecnique had to be varied to meet each individual case, but the general outline as given was adhered to.

I would call attention to the Y shaped incision for decompression operations. It gives a larger and better exposure than either the straight, curved, or horseshoe incision.

In removing the block of bone the old friend "The Rongier" was not used. As a general rule it is safe to go out 1/2 inch beyond the bone opening or at least far enough to be sure that we are over intact and uninjured dura.

The after care is most important. First absolute rest, quiet, dark room and careful handling and dressing.

The cases ordinarily seen in civil practice differ but little from war injuries. They should be handled in the same manner and with better equipment and sanitation, sterilization, hospitalization, etc., the results should be even better.

